# Blood Pressure Variability and Outcome in Traumatic Brain Injury: A Propensity Score Matching Study

**DOI:** 10.5811/westjem.2022.6.55549

**Published:** 2022-08-19

**Authors:** Quincy K. Tran, Hannah Frederick, Cecilia Tran, Hammad Baqai, Tucker Lurie, Julianna Solomon, Ayah Aligabi, Joshua Olexa, Stephanie Cardona, Uttam Bodanapally, Gary Schwartzbauer, Jessica Downing

**Affiliations:** * University of Maryland School of Medicine, Baltimore, Maryland; † University of Maryland School of Medicine, Research Associate Program in Emergency Medicine & Critical Care, Department of Emergency Medicine, Baltimore, Maryland; ‡ The R. Adams Cowley Shock Trauma Center, University of Maryland School of Medicine, Baltimore, Maryland; § University of Maryland School of Medicine, Department of Emergency Medicine, Baltimore, Maryland; ¶ Wellspan York Hospital, Department of Emergency Medicine, York, Pennsylvania; || University of Maryland School of Medicine, Department of Neurosurgery, Baltimore, Maryland; ** Mount Sinai Hospital, Department of Critical Care Medicine, York, New York; †† The R. Adams Cowley Shock Trauma Center, University of Maryland School of Medicine, Baltimore, Program in Trauma/Surgical Critical Care, Baltimore, Maryland

## Abstract

**Introduction:**

Patients with tIPH (used here to refer to traumatic intraparenchymal hemorrhagic contusion) or intraparenchymal hemorrhage face high rates of mortality and persistent functional deficits. Prior studies have found an association between blood pressure variability (BPV) and neurologic outcomes in patients with spontaneous IPH. Our study investigated the association between BPV and discharge destination (a proxy for functional outcome) in patients with tIPH.

**Methods:**

We retrospectively reviewed the charts of patients admitted to a Level I trauma center for ≥ 24 hours with tIPH. We examined variability in hourly BP measurements over the first 24 hours of hospitalization. Our outcome of interest was discharge destination (home vs facility). We performed 1:1 propensity score matching and multivariate regressions to identify demographic and clinical factors predictive of discharge home.

**Results:**

We included 354 patients; 91 were discharged home and 263 to a location other than home. The mean age was 56 (SD 21), 260 (73%) were male, 22 (6%) were on anticoagulation, and 54 (15%) on antiplatelet therapy. Our propensity-matched cohorts included 76 patients who were discharged home and 76 who were discharged to a location other than home. One measure of BPV (successive variation in systolic BP) was identified as an independent predictor of discharge location in our propensity-matched cohorts (odds ratio 0.89, 95% confidence interval 0.8–0.98; P = 0.02). Our model demonstrated good goodness of fit (P-value for Hosmer-Lemeshow test = 0.88) and very good discriminatory capability (AUROC = 0.81). High Glasgow Coma Scale score at 24 hours and treatment with fresh frozen plasma were also associated with discharge home.

**Conclusion:**

Our study suggests that increased BPV is associated with lower rates of discharge home after initial hospitalization among patients with tIPH. Additional research is needed to evaluate the impact of BP control on patient outcomes.

## INTRODUCTION

Traumatic brain injury (TBI) is the leading cause of disability and neurologic impairment among trauma survivors in the United States.^1^ Several prior studies have demonstrated that patients who experienced even mild TBI face persistent neurologic symptoms, functional limitations, and decreased quality of life for years after the injury, while those with more severe TBI—including tIPH (which we use here to refer to traumatic intraparenchymal hemorrhage or hemorrhagic contusion)—have higher mortality rates as well.^2^–^5^ Moreover, despite a decrease in the incidence of TBI, improved survival rates have resulted in an increasing population of TBI survivors with persistent neurologic sequelae.^6^ Much of the existing literature highlights the role of social, demographic, or injury-specific factors in determining outcomes associated with TBI: older age at the time of injury; the presence of preexisting medical and psychiatric comorbidities, low initial Glasgow Coma Scale (GCS) score; and initial injury severity have all been associated with increased mortality and poorer functional outcomes.^7^–^13^ Although each of these factors can serve as important markers of risk, as well as epidemiological and public health tools, none is directly impacted by initial treatment in the inpatient setting. There is limited evidence regarding the impact of initial resuscitation interventions and goals on outcomes among TBI patients.

The treatment of patients with tIPH is primarily supportive, focusing on the prevention of “secondary injury” caused by hypotension, intracranial hypertension, and hypoxia.^14^ Prior studies have demonstrated an association between blood pressure variability (BPV) and poor outcomes among patients with spontaneous IPH (sIPH), who face similarly high rates of morbidity and mortality after the initial insult as patients with tIPH.^15^–^17^ In the first hour after TBI, patients are thought to experience impaired cerebral autoregulation, which limits their ability to maintain cerebral blood flow in the face of BPV and has been associated with poor neurologic outcomes.^15^ Although prior studies have suggested that systolic hypotension is associated with worse neurologic outcomes among patients with TBI, 16–19 there is no current literature investigating the impact of BPV on neurologic outcomes in this population.

The term BPV refers to the magnitude of change in blood pressure over a defined period of time. There are three main forms of BPV: 1) successive variation in systolic BP (SBP_SV_); 2) SD in SBP (SBP_SD_); and 3) coefficient variation in SBP (SBP_CV_). Each of these three measurements examine the change in blood pressure in a slightly different way. The SBP_SV_ is the average absolute difference between successive BP measurements and represents the “steepness” of change between sequential measurements of SBP.^20^–^23^ The SBP_SD_ and SBP_CV_ measure variation of BPV over time and capture how tightly the BP is controlled.^21^–^23^ Blood pressure variability can be calculated using either SBP, diastolic BP, or mean arterial pressure (MAP); here we have focused on SBP, as blood pressure targets for patients with traumatic brain injury and intracranial hemorrhage at our institution focus on SBP.

**Population Health Research Capsule**

What do we already know about this issue?

*Blood pressure variability has been associated with worse outcomes in spontaneous intracranial hemorrhage and stroke; its impact in traumatic brain injury (TBI) is unknown.*

What was the research question?

*Does blood pressure variability impact discharge destination for patients with TBI?*

What was the major finding of the study?

*For patients with traumatic brain injury, blood pressure variability is associated with lower rates of discharge home (OR = 0.89) compared to matched controls.*

How does this improve population health?

*This suggests that clinicians may be able to improve functional outcomes in TBI by reducing blood pressure variability.*

In this study, we used propensity score matching to investigate the association between systolic BPV within the first 24 hours of admission with discharge destination (used in part as a proxy for functional outcome at hospital discharge) among patients admitted to a Level I trauma center with tIPH. While this investigation did not examine the degree to which the impact of BPV on neurologic outcomes is modifiable (or even BPV itself), our aim was to identify risk factors with the potential to be clinically modifiable to inform future research on initial care for TBI patients.

## METHODS

### Study Design and Patient Selection

This was a retrospective study of adult patients (age ≥18 years) who sustained isolated TBI with tIPH on computed tomography (CT) and were admitted to our institution for at least 24 hours between January 1, 2018–December 31, 2019. We included patients admitted to intensive care units (ICU), as well as those admitted to intermediate care or “step-down” units. We excluded patients who had only one CT during their hospitalization, as we were interested in the association between hematoma progression (here referring to volume expansion) and functional outcomes at discharge. We excluded patients with traumatic extraparenchymal (epidural, subdural, or subarachnoid) intracranial hemorrhage because the management for these conditions, when severe, is often surgical and thus less reliant on supportive measures. We did not exclude patients who underwent surgical management (namely, external ventricular drain placement or craniectomy) for intracranial hypertension as the result of tIPH. We also excluded patients with other concomitant traumatic injuries or with incomplete records. Our study was approved by our institutional review board (HP-00089624).

### Study Setting

This study was conducted at an academic Level I regional trauma center in Maryland. Most patients admitted at our institution are transported directly from the field, although we also accept transfers from several regional non-trauma centers. Upon arrival, patients are immediately evaluated by the trauma and neurosurgical teams. Patients with intracranial injury identified on initial CT are typically monitored with a repeat CT within 24 hours of admission; those without cognitive impairment or significant change in tIPH on repeat CT are often discharged home within 24 hours of admission, while those with cognitive impairment or worsening tIPH are admitted.

### Independent Variables

We obtained demographic and clinical data for each patient from our institution’s electronic health records. Data included age, gender, treatment with anticoagulation (AC) or antiplatelet therapy, past medical history, mechanism of injury, GCS score, initial laboratory values, and all recorded SBPs within 24 hours of admission to our institution. Our institution requires that BP be documented for all patients admitted for TBI at least once per hour for the first 24 hours of admission. This protocol begins in our trauma bay at the time that any intracranial hemorrhage is identified and is continued in the ICU or an intermediate care unit. Using these values, we calculated SBP_SV_, SBP_SD_, and SBP_CV_. We also identified the lowest SBP and the highest SBP during the first 24 hours of admission.

We collected data regarding patient’s mode of arrival to our trauma center, classified as “direct admission,” defined presentation to our trauma center directly from the field via emergency medical services, or “transfer” from an outside hospital or ED. We included data regarding medical interventions provided during the first 24 hours of hospital admission, including the placement of arterial lines and administration of vasopressors or antihypertensive medications, as well as hematoma and contusion volumes, which were calculated using the ABC/2 method.^24^ The placement of arterial lines was left to the discretion of the treating physicians; it is our typical practice to place arterial lines in patients with head injury who require titration of medications to maintain their blood pressure within a target range (typically, MAP >65 and SBP <180). Those who maintain their SBP within target without intervention are often not given arterial lines. We used a standardized Excel spreadsheet (Microsoft Corporation, Redmond, WA) to record data.

### Outcomes

Our primary outcome of interest was discharge destination. For the majority of our analysis, this was dichotomized as “discharged home” or “not discharged home.” Prior studies have demonstrated a correlation between discharge home from acute hospitalization and better long-term neurologic outcomes.^7^,^8^,^25^ At our institution, TBI patients participate in a rigorous multidisciplinary evaluation process to determine discharge disposition; this process begins shortly after hospital admission. All patients with TBI who were admitted for longer than 24 hours are evaluated by specialists in physical therapy (PT), occupational therapy (OT) and, often, speech and language pathology. Based on these specialists’ assessments, patients might be discharged home with or without services (such as PT therapy or nursing), to an acute or subacute rehabilitation center, or to a skilled nursing facility. Discharge recommendations are primarily determined by mobility level, ability to perform activities of daily living (ADL), cognitive ability, and home environment and available assistance.

Cognition is assessed using the Rancho Los Amigos Scale (RLAS) and is often the key driver of discharge recommendations among patients with TBI. The RLAS is a widely accepted tool used specifically in the assessment of patients with TBI. It characterizes patients’ level of awareness, response to and interaction with the environment (including clinicians), and emotional response and regulation, and independence. It has previously been shown to have utility in both characterizing initial injury and predicting rehabilitation needs and outcomes.^26^,^27^ Typically, RLAS scores of 1–3 are considered to reflect coma emergence at the time of discharge planning, and often result in a recommendation of discharge to subacute rehabilitation. Scores of 4–6 indicate a clinical state characterized by confusion and inappropriate behavior, often recommended for discharge to acute TBI rehabilitation. An RLAS score >7 indicates purposeful interaction and appropriate behavior; these patients can often be discharged home. If a patient with baseline impairment (RLAS <7) is determined to be at their neurologic baseline, they will often be discharged home (or back to their prior facility) regardless of RLAS score, if a safe home setup has been confirmed.

The Activity Measure for Postacute Care (AM-PAC) “6-Clicks” Basic Mobility and Daily Activity score—which measure basic mobility, applied cognition, and independence in daily activity, and has been previously validated as a predictor of discharge destination for a variety of disease states—is also integrated into final discharge recommendations, primarily for patients with higher RLAS scores who may be appropriate for discharge home.^28^ The AM-PAC “6-Clicks” scores are calculated at each PT and OT session and used to monitor progress and guide discharge planning.

Patients discharged shortly after 24 hours of observation receive the same evaluation by PT and OT and are generally determined to have no or only mild deficits or to be at their neurologic baseline, and appropriate for discharge home or to the same facility from where they originally presented. Patients are only eligible for discharge at or shortly after 24 hours if CT imaging remains stable and no intravenous or as needed medications are required for pain, behavior, or blood pressure control.

### Data Collection and Management

All research team members were trained in data collection by the principal investigator and senior investigators. Researchers began by collecting data from patient charts in sets of 10; their data was then compared to those collected by a senior investigator until an interrater agreement of at least 90% was achieved. A senior investigator subsequently checked 10% of each researcher’s data randomly to ensure persistent agreement. Researchers collected data in segments to reduce bias; for example, a researcher collecting BP measurements would not have access to discharge disposition, and vice versa.

### Sample Size Calculation

Given the lack of prior studies regarding BPV in patients with tIPH, we based our sample size calculation on a previous study by Tuteja et al that compared BPV among patients with sIPH.^29^ Tuteja et al demonstrated a difference of 12 millimeters of mercury (mm Hg) (SD 20 mm Hg) in SBP_SV_ between survivors and non-survivors. Based on this finding, we estimated a sample size of at least 90 patients (45 in each group) would be required to detect the same effect size with a power of 80% and α = 0.05. Given the difference in pathology between our patient population and that investigated by Tuteja et al, we aimed to collect data from as many patients as possible during our study period to improve the accuracy of our analysis.

### Data Analysis

We presented our patients’ data using descriptive analyses with mean (±SD) or median (interquartile range), as appropriate according to the distribution of the data. We analyzed continuous data using the *t* test or the Mann-Whitney *U* test, as appropriate, and categorical data via the chi-square or Fisher’s exact test as indicated. We constructed logistic regression models to identify patients with similar demographic and clinical backgrounds and calculate each patient’s propensity score for the outcome of discharge home. We performed 1:1 propensity score matching without replacement and used a stricter caliper width of 0.1, instead of the recommended width of 0.2,^30^ for the logistic regression for propensity score matching for discharge home. We selected a priori the following patient characteristics to construct the logistic regression for propensity score:

- Age
- GCS score at admission
- Hematoma and/or contusion volume at admission
- Active AC therapy at the time of injury
- Type of hemorrhage
- Intubation during hospitalization
- External ventricular drain or craniectomy during hospitalization.

We subsequently used stepwise multivariable logistic regressions to identify associations between demographic and clinical factors and outcomes among patients in the unmatched and matched groups. The independent variables were selected prior to statistical analysis and are reported in Appendix 1. Independent variables identified as significant by our stepwise multivariable logistic regressions were reported as odds ratio (OR), with 95% confidence interval (CI) when available. We assessed the goodness of fit of our regressions via the Hosmer-Lemeshow test, with *P*-value ≥0.05 indicating good fit for the independent variables. We also assessed the collinearity of our independent variables using the variance inflation factor (VIF). Any factors with VIF >5 were considered to have collinearity and were removed from the regression models. We further assessed the discriminatory capability of our regressions using the area under the receiver operating characteristic curve (AUROC), with a regression model having AUROC of 1 suggesting perfect discriminatory capability and AUROC of 5 suggesting poor discriminatory capability between the dichotomous outcomes.

Ordinal logistic regression was also performed to assess associations of demographic and clinical factors with discharge destination. We used the same independent variables as listed in Appendix 1. We reported the results of the ordinal regression with the coefficients, 95% CI, and the Somers’ delta and Goodman-Kruskal gamma tests for goodness of fit. For ordinal regressions, discharge destinations were classified as 0 (home), 1 (acute rehabilitation), 2 (skilled nursing facility), and 3 (hospice or in-hospital death). Positive coefficients indicate that as the independent variable increases, lower ranked discharge destinations (such as discharge home) become more likely, while negative coefficients indicate that as the variable increases, higher ranked discharge destinations (such as skilled nursing facility or death) become more likely. Values for the Somers’ delta and Goodman-Kruskal gamma tests range between −1 and 1. Values approaching maximum indicates good predictive ability.

We used XLSTAT to perform our propensity score matching (https://www.xlstat.com/en/; Addinsoft, Paris, France). All other statistical analyses were performed with Minitab version 19 (Minitab LLC, State College, PA). All analyses with two-tailed *P*-value ≤ 0.05 were considered statistically significant.

## RESULTS

**Figure 1:**
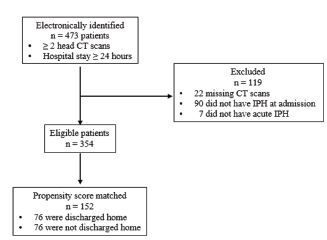
Patient selection diagram. *CT*, computed tomography; *IPH*, intraparenchymal contusion or hemorrhage.

We electronically identified 473 patients; a total of 354 patients (unmatched) were included in our logistic regression for propensity score matching (Figure 1). The mean age was 56 (SD 21), and 260 (73%) were male. Twenty-two patients (6%) were on AC therapy, and 54 (15%) on antiplatelet therapy. There were 234 patients (66%) who had contusions alone. Our propensity-matching analysis matched 76 patients who were discharged home with 76 patients who were discharged to a location other than home. In the unmatched cohorts, patients who were discharged home were younger and less likely to be on AC or antiplatelet therapy (Table 1). They were less likely to have received hyperosmolar therapy, blood transfusion, or craniectomy, were less likely to have had an arterial line placed or to have been treated with vasopressors or antihypertensives, and were less likely to require ICU admission (Table 2). Patients who were discharged home had significantly lower BPV and higher RLAS and AM-PAC “6-Clicks” scores (Table 3). There were no significant differences between the propensity-matched cohorts with respect to the demographic characteristics we examined (Table 1), interventions within 24 hours of admission, including administration of vasopressors or antihypertensive medications (Table 2). There was no difference with respect to 24-hour BPV (Table 3). Patients who were discharged home had higher median GCS scores at 24 hours and on hospital day 5 and higher AM-PAC “6 Clicks” scores and RLAS scores (Table 3).

### Primary Outcome: Discharge Destination

#### Unmatched Cohorts

Stepwise multivariable logistic regression of the unmatched cohorts reported seven independent variables as significant for the regression (Table 4). SBP_SV_ was considered important for the regression but did not show a statistically significant association with discharge home (OR 0.94, 95% CI 0.88–1.009; *P* = 0.09). No other components of BPV were identified as having an association with our primary outcome. This model showed good fit of the independent variables (*P* value for Hosmer-Lemeshow test = 0.84) and very good discriminatory capability (AUROC = 0.91). All factors had low variance inflation factor (VIF), demonstrating no collinearity.

#### Propensity-Matched Cohorts

**Figure 2:**
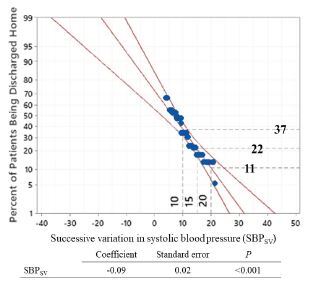
Probit and logit analysis demonstrating the probability of discharge home according to SBPSV (mm Hg). *SBPSV*, successive variation in systolic blood pressure.

We performed stepwise multivariable logistic regressions of the matched cohorts and identified six independent variables as important (Table 4). SBP_SV_ (OR 0.89, 95% CI 0.8–0.98, *P* = 0.02) was significantly associated with discharge home. A SBP_SV_ of 10 mm Hg over the first 24 hours of hospitalization was associated with a 37% likelihood of discharge home, while a SBP_SV_ of 20 mm Hg over the same timeframe was associated with an 11% likelihood of discharge home (Figure 2). There was goodness of fit of the independent variables (*P* value for Hosmer-Lemeshow test = 0.88) and very good discriminatory capability (AUROC = 0.81). The included variables did not show collinearity; all had low VIF. In addition, high GCS score at 24 hours (OR 1.5, 95% CI 1.2–1.9; *P* = 0.001) was associated with higher likelihood of discharge home.

Our ordinal logistic regressions identified six variables significantly associated with discharge destination (Table 5). Among the matched cohorts, higher SBP_SV_ was associated with lower likelihood of discharge to destinations requiring higher levels of independence, such as home or acute rehabilitation (OR 0.90, 95% CI 0.82, 0.99; *P* = 0.05). No other measures of BPV were significantly associated with discharge location.

## DISCUSSION

Our study identified few independent factors that were associated with discharge home (a proxy for good functional outcome at hospital discharge) among patients who sustained isolated TBI with IPH. Of the independent variables, one component of BPV, the SBP_SV_, was significantly associated with likelihood of discharge home among the propensity-matched cohorts.

Blood pressure has previously been considered an important predictor of outcomes among patients with neurologic injury. Traditionally, researchers have focused on absolute blood pressure values. Rasmussen et al, for example, demonstrated an association between both hypertension (MAP >90 mm Hg) and hypotension (MAP <70 mm Hg) and poor neurologic outcomes among patients with ischemic strokes treated with endovascular therapy.^31^ Brenner et al found that even mild hypotension (SBP <120 mm Hg) was associated with poor neurologic outcomes in patients with severe TBI.^32^ Systolic hypertension has similarly been associated with both hematoma growth and poor outcomes in patients with sIPH,^33^ for whom early control of SBP has been recommended as a mainstay of supportive care.^34^ Blood pressure variability has been shown to have important impacts on the neurologic outcomes of patients with both ischemic strokes and sIPH.^20^,^22^,^23^,^33^,^35^–^37^ To our knowledge, ours is the first investigation into the impact of BPV specifically among patients with tIPH.

Our findings overlap with those previously demonstrated among patients with spontaneous (most often hypertensive) hemorrhage. Tanaka et al conducted a multicenter prospective observational study to investigate the impact of systolic BPV during the initial 24 hours after intracranial hemorrhage.^22^ All included patients with SBP > 160 mm Hg received intravenous nicardipine during this period. We found that both increased SBP_SD_ and SBP_SV_ were associated with neurologic deterioration (defined as a decrease in GCS score of 2 or more points or an increase in National Institutes of Health Stroke Scale^38^ by 4 or more points), as well as that increased SBP_SV_ was associated with unfavorable neurologic outcomes at three months. Chung et al reported a similar association of BPV (including the three key components studied here: SBP_SV_, SBP_SD_, and SBP_CV_) with poor neurologic outcome at three months in both of what they describe as the hyperacute (0 to 4–6 hours) and acute (0 to 24–26 hours) stages among 386 patients with intracerebral hemorrhage.^23^

Our results vary from those reported in these studies in that, in our study SBP_SV_ was the only BPV component associated with neurologic outcomes. While SBP_SD_ and SBP_CV_ reflect BPV over the entire reported time period, SBP_SV_ is related to the sequence of BP measurements and thus more precisely reflects variation between measurements. In this study, SBP_SV_ provides a measurement of variability on an hourly basis. Our findings suggest that steeper “swings” in SBP may be more important than overall variation in patients with tIPH, whereas both types of variability are relevant for patients with sIPH. This difference may be reflective of the etiology of the initial injury, as hypertension is one of the most common risk factors for sIPH.^39^ Preexisting cardiovascular disease may predispose patients with sIPH to increased sensitivity to even slow changes in BP relative to those with TBI.

In addition, we used a different endpoint to reflect neurologic status than the majority of prior investigations into BP and BPV in neurologic insult, which primarily rely on the modified Rankin scale,^38^ Glasgow Outcome Scale,^41^ or Glasgow Outcome Scale-Extended^42^ several months after the insult. Each of these measures provides insight into patients’ level of functioning and disability, particularly with respect to ADL. Several components of post-TBI care—most notably PT, OT, and cognitive therapy—have important impacts on patients’ ultimate neurologic recovery and functional status.

By focusing on discharge destination, we hoped to highlight an outcome that is 1) more directly related to in-hospital care, particularly in the early stages of the patient’s injury; 2) reflective of the patient’s functional status and recovery trajectory; and 3) in itself meaningful to both patients and the healthcare system, where discharge planning has taken on additional importance due to increased concerns over hospital-acquired infections and other complications, as well as length of stay and inpatient reimbursement. Because discharge destination is largely driven by in-depth assessments by rehabilitation specialists using a variety of validated scoring tools as well as individualized assessment of each patient’s status and capabilities, we expect it adequately meets these requirements. Prior studies have demonstrated that demographic, social, and nonmodifiable clinical factors (such as injury severity) play an important role in discharge destination.^9^–^11^,^43^–^46^ To our knowledge, this is the first paper to identify a potentially modifiable risk factor for discharge destination.

### Implications for Future Research

Unlike many predictors of neurologic outcomes among patients with neurologic injury, BPV is a possibly modifiable risk factor with the potential to be directly impacted by medical care. This is true in both traumatic and spontaneous injuries, including those examined in our unmatched analysis. Prior studies have demonstrated that close control of hypertension and BPV using intravenous agents such as nicardipine may improve outcomes among patients with sIPH. Our results support a similar association among patients with tIPH, although further research is needed to specifically investigate the role and impact of BP control, rather than BPV alone, among patients with tIPH. Our findings further suggest that management of tIPH should emphasize slow and steady control that avoids rapid swings in BP.^22^,^23^

It may be the case that greater BPV reflects the severity of the underlying injury as well as contributes to poorer outcomes. A prior study demonstrated that dynamic cerebral autoregulation—the mechanism by which cerebral blood flow is quickly restored in response to rapid changes in perfusion pressure—may be impaired in brain tissue affected by large infarcts.^43^ Intracerebral hemorrhage has also been associated with decreased “baroreflex sensitivity,” resulting in greater BPV in patients experiencing intracerebral hemorrhage when compared to healthy controls; this decreased baroreflex sensitivity has been associated with increased likelihood of hematoma expansion and poor neurologic outcomes.^44^ Although evidence to date suggests that increased BPV impacts outcomes, BPV may itself reflect impairment in cerebral autoregulation due to more severe initial injury. Further studies are needed to examine the degree to which BPV, and its impact on neurologic outcomes, are truly modifiable.

## LIMITATIONS

In the setting of critical illness or injury, hemodynamics often change rapidly. Our results highlight the importance of the speed of this change, as well as its magnitude, for neurologic outcomes in patients with TBI. However, we were able to analyze only BPs recorded in patients’ medical records, which often occurred on an hourly basis. Although we captured all BP measurements recorded within 24 hours of patient arrival, these values may not fully or accurately represent patients’ hemodynamic status. Similarly, although we have presented data regarding the use of vasopressors and antihypertensive medications, we were unable to reliably determine when these medications were started relative to the BP measurements we analyzed, and thus the impact these medications had on BP variability. Finally, although we attempted to minimize biases in our study, some forms of bias, likely due to the selection of independent variables, may have existed. As a result, we observed that SBP_SV_ was statistically significant in the matched cohorts, but not in the unmatched cohorts, where we expect the relatively small effect size of each mm Hg was likely masked by those of other variables.

## CONCLUSION

Our study suggests that increased blood pressure variability, and specifically successive variation in systolic blood pressure, is associated with lower rates of discharge home after initial hospitalization among patients with traumatic intraparenchymal hemorrhage or hemorrhagic contusion. Age and hematoma/contusion volume at admission were also associated with lower rates of discharge home among the unmatched cohorts. Further research is needed to investigate the impact of medical control of BP on discharge destination and neurologic outcomes.

## Supplementary Information



## Figures and Tables

**Table 1:** Characteristics of 354 patients with traumatic intraparenchymal contusion or hemorrhage by discharge location, matched and unmatched cohorts.

	All patients (N = 354)	Unmatched cohorts			Propensity-matched cohorts		

		Discharged home (n = 91)	Not discharged home (n = 263)	*P*	Discharged home (n = 76)	Not discharged home (n = 76)	*P*
Demographics							
Age, mean (SD)	56 (21)	47 (19)	58 (21)	< 0.001	50 (19)	50 (20)	0.91
Male, n (%)	260 (73)	72 (79)	188 (71)	0.15	62 (82)	56 (74)	0.24
BMI, mean (SD)	26 (5)	25 (5)	26 (5)	0.50	25 (5)	25 (5)	0.91
Direct admission, n (%)	262 (74)	63 (69)	199 (76)	0.22	53 (70)	62 (82)	0.09
Transfer, n (%)	91 (26)	27 (30)	64 (24)	0.31	23 (30)	14 (18)	0.09
Past medical history, n (%)							
Any AC therapy	22 (6)	1 (1)	21 (8)	0.019	1 (1)	2 (3)	0.56
Any antiplatelet therapy	54 (15)	7 (8)	47 (18)	0.02	7 (9)	10 (13)	0.44
Hypertension	135 (38)	30 (33)	105 (40)	0.23	29 (38)	25 (33)	0.49
Diabetes mellitus	45 (13)	7 (8)	38 (14)	0.10	7 (9)	10 (13)	0.44
Type of injury, n (%)							
Hemorrhage only	93 (26)	14 (15)	79 (30)	0.01	15 (20)	13 (17)	0.67
Contusion only	234 (66)	73 (80)	161 (61)	0.001	58 (76)	59 (78)	0.84
Hemorrhage and contusion	27 (8)	4 (4)	23 (9)	0.18	3 (4)	4 (5)	0.69
Mechanism of injury, n (%)							
Fall	200 (56)	52 (57)	148 (56)	0.89	46 (61)	36 (47)	0.10
MVC	91 (26)	17 (19)	74 (28)	0.09	16 (21)	25 (33)	0.10
Other blunt trauma	43 (12)	20 (22)	23 (9)	0.001	13 (17)	11 (14)	0.65
Any penetrating trauma	7 (2)	0 (0)	7 (3)	0.11	0 (0)	2 (3)	0.15
Unknown	13 (4)	2 (2)	11 (4)	0.38	1 (1)	2 (3)	0.56
Laboratory values at admission							
Lactate, mmol/L, median (IQR)	2.5 (1.6–3.6)	2.2 (1.6–3.4)	2.6 (1.6–2.2)	0.23	2.3 (1.5–3.6)	2.3 (1.7–3.7)	0.59
INR, mean (SD)	1.1 (0.4)	1.0 (0.1)	1.2 (0.5)	< 0.001	1.0 (0.2)	1.1 (0.2)	0.02

*AC*, anticoagulation; *BMI*, body mass index; *INR*, international normalized ratio; *IQR*, interquartile range; *MVC*, motor vehicle collision.

**Table 2:** Comparison of interventions provided in the first 24 hours of hospitalization by discharge location, matched and unmatched cohorts.

	All patients (N = 354)	Unmatched cohorts			Propensity-matched cohorts		

		Discharged home (n = 91)	Not discharged home (n = 263)	*P*	Discharged home (n = 76)	Not discharged home (n = 76)	*P*
Mechanical ventilation, n (%)	202 (57)	30 (33)	172 (65)	< 0.001	33 (43)	33 (43)	0.99
IV crystalloids, n (%)	310 (88)	81 (89)	229 (87)	0.62	69 (91)	67 (88)	0.59
24-h crystalloid administration, mL, mean (SD)	1927 (1772)	1532 (1238)	2063 (1906)	0.003	1798 (1334)	1696 (1597)	0.67
Fluid balance at 24-h, mL, mean (SD)	1236 (2188)	864 (1880)	1365 (2274)	0.04	931 (1965)	1192 (1685)	0.38
Any AED, n (%)	86 (25)	19 (21)	67 (26)	0.34	13 (17)	16 (21)	0.53
Phenytoin, n (%)	73 (21)	16 (18)	57 (22)	0.40	11 (14)	14 (18)	0.51
Levetiracetam, n (%)	13 (4)	3 (3)	10 (4)	0.82	2 (3)	2 (3)	0.99
Any hyperosmolar agent, n (%)^a^	118 (33)	11 (12)	107 (41)	< 0.001	13 (17)	11 (14)	0.65
3% saline, n (%)	108 (31)	11 (12)	97 (37)	< 0.001	13 (17)	11 (14)	0.65
Mannitol, n (%)	10 (3)	0 (0)	10 (4)	0.06	0 (0)	1 (1)	0.99
Invasive BP monitoring, n (%)	151 (43)	15 (15)	136 (53)	< 0.001	13 (17)	23 (30)	0.09
Any antiHTN medication, n (%)	121 (34)	20 (22)	101 (38)	< 0.001	21 (26)	21 (27)	0.99
AntiHTN infusion, n (%)	60 (17)	4 (4)	56 (21)	< 0.001	4 (5)	4 (5)	0.99
Any vasopressor, n (%)	113 (32)	17 (19)	96 (37)	<0.001	16 (21)	21 (27)	0.45
Any blood products, n (%)^a^	106 (30)	10 (11)	96 (37)	< 0.001	10 (13)	15 (20)	0.27
pRBC, n (%)	57 (16)	4 (4)	53 (20)	< 0.001	4 (5)	7 (9)	0.34
FFP, n (%)	11 (3)	2 (2)	9 (3)	0.56	2 (3)	1 (1)	0.56
Platelets, n (%)	38 (11)	4 (4)	34 (13)	0.02	4 (5)	7 (9)	0.34
EVD, n (%)	80 (23)	3 (3)	77 (29)	< 0.001	6 (8)	4 (5)	0.51
Opening pressure, cm H_2_O, mean (SD)	15 (8)	11 (8)	15 (8)	0.61	11 (5)	18 (9)	0.20
Craniectomy, n (%)	53 (15)	3 (3)	50 (19)	< 0.001	5 (7)	5 (7)	0.99
ICU admission, n (%)	214 (60)	36 (40)	178 (68)	< 0.001	31 (41)	43 (56)	0.07

a Patients could receive more than one product.

*IV*, intravenous; *mL*, milliliter; *cm*, centimeter; *AED*, antiepileptic drug; *antiHTN*, anti-hypertensive; *BP*, blood pressure; *EVD*, external ventricular drain; *FFP*, fresh frozen plasma; *ICU*, intensive care unit; *IV*, intravenous; *pRBC*, packed red blood cells.

**Table 3:** Comparison of clinical characteristics by discharge location, matched and unmatched cohorts.

	All patients (N = 354)	Unmatched cohorts			Propensity-matched cohorts		

		Discharged home (n = 91)	Not discharged home (n = 263)	*P*	Discharged home (n = 76)	Not discharged home (n = 76)	*P*
BPV							
SBPmax, mean (SD)	178 (31)	165 (24)	182 (32)	< 0.001	170 (24)	173 (27)	0.38
SBPmin, mean (SD)	91 (28)	100 (26)	89 (28)	< 0.001	100 (26)	95 (29)	0.32
SBPmax–min, mean (SD)	87 (41)	66 (32)	94 (41)	< 0.001	70 (31)	78 (40)	0.16
SBPSV, mean (SD)	14 (6)	11 (4)	15 (6)	< 0.001	12 (4)	13 (6)	0.09
SBPSD, mean (SD)	17 (7)	13 (7)	18 (7)	< 0.001	14 (7)	15 (7)	0.47
SBPCV, mean (SD)	16 (6)	13 (5)	17 (7)	< 0.001	13 (5)	15 (7)	0.11
Hematoma/contusion volume							
Initial (cm^3^), mean (SD)	0.5 (0.4)	1.3 (2.2)	9.1 (23)	< 0.001	3 (4)	4 (11)	0.07
Progression, n (%)	160 (45)	40 (44)	120 (46)	0.78	35 (46)	31 (41)	0.51
GCS score, median (IQR)							
At admission	13 (7–14)	14 (12–15)	11 (6–14)	< 0.001	14 (10–15)	14 (10–14)	0.71
At 24 hours	11 (7–14)	15 (14–15)	10 (7–14)	< 0.001	14 (13–15)	13 (10–15)	< 0.001
Functional assessments, mean (SD)							
RLAS	5.3 (1.8)	6.4 (1.5)	4.9 (1.7)	< 0.001	6.4 (1.6)	5.7 (1.2)	< 0.001
AM-PAC “6-Clicks” score	15 (6)	20 (4)	12 (5)	< 0.001	20 (4)	15 (4)	< 0.001
Discharge disposition, n (%)							
Home	91 (26)	91 (100)	NA	NA	76 (100)	NA	NA
Acute rehabilitation	167 (47)	NA	167 (63)	NA	NA	53 (70)	NA
Skilled nursing home	34 (10)	NA	34 (13)	NA	NA	14 (18)	NA
Hospice/death	52 (15)	NA	52 (20)	NA	NA	6 (8)	NA
Other	10 (3)	NA	10 (4)	NA	NA	3 (4)	NA

*AM-PAC*, Activity Measure for Postacute Care; *BPV*, blood pressure variability; *GCS*, Glasgow Coma Scale; *IQR*, interquartile range; *NA*, not applicable; *RLAS*, Rancho Los Amigos Scale; *SBPCV*, coefficient variation in systolic blood pressure; *SBPSD*, SD in systolic blood pressure; *SBPSV*, successive variation in systolic blood pressure; *SBPmax*, maximum systolic blood pressure; *SBPmin*, minimum systolic blood pressure.

**Table 4:** Results of stepwise multivariable logistic regressions to measure associations between patient characteristics, clinical course, and discharge location, matched and unmatched cohorts.

Variable^a^	Unmatched cohorts^b^				Propensity-matched cohorts^c^			

	OR	95% CI	*P*	VIF	OR	95% CI	*P*	VIF
Age	0.95	0.93–0.97	0.001	1.5	NS	NS	NS	NS
SBPSV	0.94	0.88–1.009	0.09	1.1	0.89	0.8–0.98	0.02	1.7
Hematoma/contusion volume at admission	0.90	0.83–0.98	0.03	1.1	0.92	0.8–1.01	0.11	1.2
INR at admission	NS	NS	NS	NS	0.03	0.001–0.6	0.02	1.6
Direct admission	0.29	0.13–0.69	0.005	1.4	0.14	0.05–0.39	0.001	1.4
GCS score at 24 hours	1.5	1.3–1.8	0.001	1.2	1.5	1.2–1.9	0.001	2.7
FFP transfusion	NS	NS	NS	NS	52	3.7–50+	0.003	2.0
Mechanism of injury: MVC	0.33	0.13–0.82	0.02	1.3	NS	NS	NS	NS
EVD	0.23	0.06–0.93	0.04	1.1	NS	NS	NS	NS

a Only variables considered significant for the regression were reported.

b Hosmer-Lemeshow test: degrees of freedom 8, chi-square 4, P = 0.84; AUROC = 0.91.

c Hosmer-Lemeshow test: degrees of freedom 8, chi-square 4, P = 0.88; AUROC = 0.81.

*AUROC*, area under the receiver operating characteristic curve; *EVD*, external ventricular drain; *FFP*, fresh frozen plasma; *GCS*, Glasgow Coma Scale; *INR*, international normalized ratio; *MVC*, motor vehicle collision; *EVD*, external ventricular drain; *NS*, not significant; *OR*, odds ratio; *SBPSV*, successive variation in systolic blood pressure; *VIF*, variance inflation factor.

**Table 5:** Results of ordinal logistic regression to measure the association between patients’ characteristics, clinical course, and their hospital disposition. The hospital dispositions were ranked from 0 (Discharged home directly), 1 (Acute Rehabilitation), 2 (Skilled Nursing Home), 3 (Hospice or Dead). Only significant factors were reported here, in increasing order of unmatched variables’ coefficients.

Variables	Unmatched cohorts^1^				Propensity-matched cohorts^2^			

	Coefficient	OR	95% CI	*P*	Coefficient	OR	95% CI	*P*
INR at admission	−1.07	0.34	0.12, 0.97	0.04	−2.7	0.07	0.01, 0.76	0.03
EVD	−0.83	0.44	0.23, 0.82	<0.001	−1.7	0.18	0.04, 0.77	0.02
Platelet transfusion	−0.77	0.46	0.24, 0.91	0.03	−1.3	0.27	0.07, 0.99	0.05
Any craniectomy	−0.74	0.48	0.24, 0.94	0.03	NS	NS	NS	NS
Hematoma/contusion volume at admission	−0.02	0.97	0.96, 0.99	<0.001	−0.04	0.96	0.92, 0.999	0.04
SBPsv	NS	NS	NS	NS	−0.10	0.90	0.82, 0.999	0.05

1 Somer’s delta = 0.64; Goodman-Kruskal gamma = 0.64.

2 Somer’s delta = 0.50; Goodman-Kruskal gamma = 0.50.

*OR*, odds ratio; *CI*, confidence interval; *INR*, international normalized ratio; *EVD*, external ventricular drain; *SBPsv*, successive variation in systolic blood pressure.
