# Supplementary material for: Blood Pressure Variability and Outcome in Traumatic Brain Injury: A Propensity Score Matching Study
Source: West J Emerg Med. 2022 Aug 19;23(5):769–80. doi: 10.5811/westjem.2022.6.55549 (PMC9541979; doi:10.5811/westjem.2022.6.55549)
Supplement: Supplementary file 1 [file wjem-23-769-s001.docx]

Appendix 1. Independent variables for stepwise multivariable logistic regressions to identify associations between demographic and clinical factors and discharge locations among patients with tIPH, in both unmatched and matched groups

| **Continuous** | **Categorical** |
| --- | --- |
| Age | Gender |
| BMI | Direct admit |
| Serum lactate | Any anticoagulation |
| INR | Any antiplatelet |
| 24 hour IVF | Hypertension |
| 24 hour fluid balance | Diabetes |
| SBPmax | Hemorrhage only |
| SBPmin | Contusion only |
| SBP_SV_ | Both hemorrhage and Contusion |
| SBP_SD_ | Fall |
| SBP_CV_ | MVC |
| First hematoma volume | Any penetrating trauma |
| GCS score at admission | Mechanical ventilation |
| GCS socre at 24 hours | Any seizure medication |
|  | Any hyperosmolar therapy |
|  | Any pRBC |
|  | Any FFP |
|  | Any platelet |
|  | Any EVD |
|  | Any craniectomy |
